# *n*-Dodecyl­ammonium bromide monohydrate

**DOI:** 10.1107/S1600536810010123

**Published:** 2010-03-24

**Authors:** Wenyan Dan, Youying Di, Donghua He, Weiwei Yang, Yuxia Kong

**Affiliations:** a College of Chemistry and Chemical Engineering, Liaocheng University, Shandong 252059, People’s Republic of China

## Abstract

In the title compound, C_12_H_28_N^+^·Br^−^·H_2_O, the ionic pairs formed by *n*-dodecyl­ammonium cations and bromide anions are arranged into thick layers; these layers are linked in a nearly perpendicular fashion [the angle between the layers is 85.84 (5)°] by hydrogen-bonding inter­actions involving the water mol­ecules. The methyl­ene part of the alkyl chain in the cation adopts an all-*trans* conformation. In the crystal structure, mol­ecules are linked by inter­molecular N—H⋯Br, O—H⋯Br and N—H⋯O hydrogen bonds.

## Related literature

Long-chain *n*-alkyl­ammonium halides are widely used as surfacta­nts (Aratono *et al.*, 1998 ▶; Tornblom *et al.*, 2000 ▶) and as models for biological membranes (Ringsdorf *et al.*, 1988 ▶). They exhibit polymorphism at room temperature: for solid-solid phase transitions in *n*–alkyl­ammonium chlorides, see: Terreros *et al.* (2000 ▶). For a related structure, see: Lundén (1974 ▶).
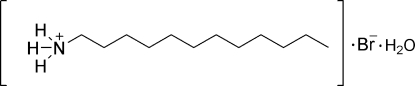


## Experimental

### 

#### Crystal data

C_12_H_28_N^+^·Br^−^·H_2_O
*M* *_r_* = 284.28
Monoclinic, 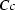

*a* = 4.7921 (5) Å
*b* = 42.810 (4) Å
*c* = 7.8573 (8) Å
β = 105.798 (2)°
*V* = 1551.0 (3) Å^3^
*Z* = 4
Mo *K*α radiation
μ = 2.63 mm^−1^
*T* = 293 K
0.42 × 0.14 × 0.06 mm

#### Data collection

Siemens SMART CCD area-detector diffractometer
Absorption correction: multi-scan (*SADABS*; Sheldrick, 1996 ▶) *T*_min_ = 0.404, *T*_max_ = 0.858
4760 measured reflections
2644 independent reflections
1665 reflections with *I* > 2σ(*I*)
*R*_int_ = 0.047

#### Refinement

*R*[*F* ^2^ > 2σ(*F* ^2^)] = 0.043
*wR*(*F* ^2^) = 0.081
*S* = 0.92
2644 reflections
145 parameters
5 restraints
H atoms treated by a mixture of independent and constrained refinement
Δρ_max_ = 0.40 e Å^−3^
Δρ_min_ = −0.24 e Å^−3^
Absolute structure: Flack (1983 ▶), 945 Friedel pairs
Flack parameter: 0.048 (19)

### 

Data collection: *SMART* (Siemens, 1996 ▶); cell refinement: *SAINT* (Siemens, 1996 ▶); data reduction: *SAINT*; program(s) used to solve structure: *SHELXS97* (Sheldrick, 2008 ▶); program(s) used to refine structure: *SHELXL97* (Sheldrick, 2008 ▶); molecular graphics: *SHELXTL* (Sheldrick, 2008 ▶) and *DIAMOND* (Brandenburg, 1998 ▶); software used to prepare material for publication: *SHELXTL*.

## Supplementary Material

Crystal structure: contains datablocks I, global. DOI: 10.1107/S1600536810010123/lx2141sup1.cif

Structure factors: contains datablocks I. DOI: 10.1107/S1600536810010123/lx2141Isup2.hkl

Additional supplementary materials:  crystallographic information; 3D view; checkCIF report

## Figures and Tables

**Table 1:** Hydrogen-bond geometry (Å, °)

*D*—H⋯*A*	*D*—H	H⋯*A*	*D*⋯*A*	*D*—H⋯*A*
N1—H1*A*⋯Br1^i^	0.89	2.53	3.380 (4)	159
N1—H1*B*⋯Br1	0.89	2.47	3.340 (4)	166
N1—H1*C*⋯O1	0.89	1.97	2.834 (7)	165
O1—H1⋯Br1^ii^	0.83 (4)	2.76 (6)	3.329 (5)	128 (6)
O1—H2⋯Br1^iii^	0.97 (4)	2.49 (5)	3.361 (5)	149 (5)

Symmetry codes: (i) 
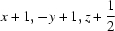
; (ii) 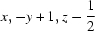; (iii) 
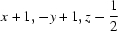
.

**Fig. 1.**
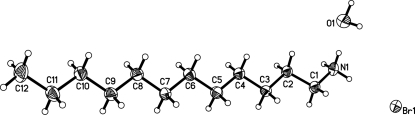
The molecular structure of the title compound with the atom numbering scheme. Displacement ellipsoids are drawn at the 50% probability level. H atoms are presented as a small spheres of arbitrary radius.

**Fig. 2.**
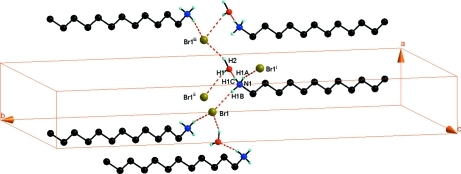
N—H···Br, N—H···O and O—H···Br interactions (dotted lines) in the crystal structure of the title compound. [Symmetry codes: (i) x + 1, - y + 1, z + 1/2; (ii) x, - y +1 , z - 1/2; (iii) x + 1, - y + 1, z - 1/2]

## supplementary crystallographic information

### Comment

Long-chain *n*-alkylammonium halides are widely used as surfactants (Aratono *et al.*, 1998; Tornblom *et al.*, 2000) and as models for biological membranes (Ringsdorf *et al.*, 1988). They exhibit polymorphism at room temperature; solid-solid phase transitions occurred in *n*-alkylammonium chlorides (Terreros *et al.*, 2000). As a part of the studies on novel potential phase transfer materials with the thermochemical properties such as *n*-alkylammonium chlorides, we report the crystal structure of the title compound (Fig. 1).

Atoms C3–C12 are coplanar in the title compound; however, atoms C2–C12 are coplanar in *n*–dodecylammonium bromide (Ludén, 1974). Although the methylene chain had the extended all–*trans* conformation, it is slightly curved in the vicinity of the ammonium group, to accommodate the hydrogen–bonding interactions. The hydrogen bonds of *n*–dodecylammonium bromide monohydrate are more stronger than that of *n*–dodecylammonium bromide because of N—H···O and O—H···Br hydrogen bonds. Only torsion angle C1–C2–C3–C4 deviates significantly from 180 °, with a value of 170.6 (5)°. The crystal packing (Fig. 2) is stabilized by intermolecular N—H···Br, N—H···O and O—H···Br hydrogen bonds (Table 1).

### Experimental

*n*–Dodecylammonium bromide monohydrate was prepared by the addition of hydrobromic acid to an ethanol solution of *n*–dodecylamine. The resulting precipitate was filtered off and recrystallized several times from chloroform. Single crystals suitable for X–ray diffraction were prepared by evaporation of a solution of the title compound in chloroform at room temperature. Analysis, calculated for C_12_H_30_BrNO (Mr = 284.28): C 50.69, H 10.64, N 4.93, O 5.63, Br 28.11%; found: C 50.67, H 10.65, N 4.91, O 5.64, Br 28.13%.

### Refinement

The reported Flack parameter was obtained by TWIN/BASF procedure in SHELXL (Sheldrick, 2008). Water molecule bound H atoms were located in difference Fourier maps and their positional parameters refined with a distance restraint [O1—H1 = 0.85 (5) & O1—H2 = 0.80 (5) Å] and a angle restraint. The H atoms of C and N atoms were positioned geometrically, with methylene C—H distances of 0.97 Å, methyl C—H distances of 0.96 Å, N—H 0.89 Å and refined as riding on their parent atoms. The*U*_iso_(H) values were set at 1.2*U*_eq_ for the methylene H atoms and at 1.5*U*_eq_ for other H atoms.

### Crystal data

**Table:**

C_12_H_28_N^+^·Br^−^·H_2_O	*F*(000) = 608
*M**_r_* = 284.28	*D*_x_ = 1.217 Mg m^−^^3^
Monoclinic, *C**c*	Mo *K*α radiation, λ = 0.71073 Å
Hall symbol: C -2yc	Cell parameters from 1266 reflections
*a* = 4.7921 (5) Å	θ = 2.9–25.0°
*b* = 42.810 (4) Å	µ = 2.63 mm^−^^1^
*c* = 7.8573 (8) Å	*T* = 293 K
β = 105.798 (2)°	Acicular, colourless
*V* = 1551.0 (3) Å^3^	0.42 × 0.14 × 0.06 mm
*Z* = 4	

### Data collection

**Table:**

Siemens SMART CCD area-detector diffractometer	2644 independent reflections
Radiation source: fine-focus sealed tube	1665 reflections with *I* > 2σ(*I*)
graphite	*R*_int_ = 0.047
Detector resolution: 10 pixels mm^-1^	θ_max_ = 27.0°, θ_min_ = 1.9°
φ and ω scans	*h* = −6→5
Absorption correction: multi-scan (*SADABS*; Sheldrick, 1996)	*k* = −54→50
*T*_min_ = 0.404, *T*_max_ = 0.858	*l* = −6→10
4760 measured reflections	

### Refinement

**Table:**

Refinement on *F*^2^	Secondary atom site location: difference Fourier map
Least-squares matrix: full	Hydrogen site location: difference Fourier map
*R*[*F*^2^ > 2σ(*F*^2^)] = 0.043	H atoms treated by a mixture of independent and constrained refinement
*wR*(*F*^2^) = 0.081	*w* = 1/[σ^2^(*F*_o_^2^) + (0.0277*P*)^2^] where *P* = (*F*_o_^2^ + 2*F*_c_^2^)/3
*S* = 0.92	(Δ/σ)_max_ < 0.001
2644 reflections	Δρ_max_ = 0.40 e Å^−^^3^
145 parameters	Δρ_min_ = −0.24 e Å^−^^3^
5 restraints	Absolute structure: Flack (1983), 945 Friedel pairs
Primary atom site location: structure-invariant direct methods	Flack parameter: 0.048 (19)

### Special details

**Table:**

Geometry. All esds (except the esd in the dihedral angle between two l.s. planes) are estimated using the full covariance matrix. The cell esds are taken into account individually in the estimation of esds in distances, angles and torsion angles; correlations between esds in cell parameters are only used when they are defined by crystal symmetry. An approximate (isotropic) treatment of cell esds is used for estimating esds involving l.s. planes.
Refinement. Refinement of F^2^ against ALL reflections. The weighted R-factor wR and goodness of fit S are based on F^2^, conventional R-factors R are based on F, with F set to zero for negative F^2^. The threshold expression of F^2^ > 2sigma(F^2^) is used only for calculating R-factors(gt) etc. and is not relevant to the choice of reflections for refinement. R-factors based on F^2^ are statistically about twice as large as those based on F, and R- factors based on ALL data will be even larger.

### Fractional atomic coordinates and isotropic or equivalent isotropic displacement parameters (Å^2^)

**Table:**

	*x*	*y*	*z*	*U*_iso_*/*U*_eq_	
Br1	0.2086 (2)	0.524590 (10)	0.38293 (16)	0.05643 (18)	
O1	0.8268 (12)	0.45062 (13)	0.1580 (6)	0.0760 (15)	
H1	0.734 (13)	0.4485 (18)	0.053 (6)	0.114*	
H2	0.993 (11)	0.4589 (15)	0.124 (9)	0.114*	
N1	0.7249 (9)	0.46990 (8)	0.4809 (6)	0.0502 (12)	
H1A	0.8831	0.4731	0.5697	0.075*	
H1B	0.6083	0.4864	0.4696	0.075*	
H1C	0.7755	0.4671	0.3810	0.075*	
C1	0.5729 (12)	0.44200 (13)	0.5176 (8)	0.0496 (17)	
H1D	0.3952	0.4392	0.4238	0.059*	
H1E	0.5212	0.4449	0.6276	0.059*	
C2	0.7567 (11)	0.41323 (10)	0.5313 (7)	0.0480 (14)	
H2A	0.9345	0.4161	0.6249	0.058*	
H2B	0.8084	0.4104	0.4213	0.058*	
C3	0.6028 (11)	0.38403 (11)	0.5696 (7)	0.0496 (14)	
H3A	0.5795	0.3855	0.6881	0.059*	
H3B	0.4108	0.3831	0.4875	0.059*	
C4	0.7635 (11)	0.35416 (10)	0.5545 (7)	0.0466 (14)	
H4A	0.7908	0.3531	0.4367	0.056*	
H4B	0.9540	0.3550	0.6382	0.056*	
C5	0.6137 (11)	0.32477 (11)	0.5877 (7)	0.0484 (14)	
H5A	0.5955	0.3254	0.7076	0.058*	
H5B	0.4194	0.3245	0.5082	0.058*	
C6	0.7625 (11)	0.29500 (11)	0.5641 (7)	0.0488 (14)	
H6A	0.9550	0.2951	0.6458	0.059*	
H6B	0.7859	0.2947	0.4453	0.059*	
C7	0.6105 (11)	0.26515 (11)	0.5920 (7)	0.0454 (13)	
H7A	0.5909	0.2651	0.7117	0.055*	
H7B	0.4168	0.2651	0.5117	0.055*	
C8	0.7621 (11)	0.23602 (11)	0.5640 (7)	0.0484 (14)	
H8A	0.9555	0.2361	0.6447	0.058*	
H8B	0.7827	0.2362	0.4446	0.058*	
C9	0.6128 (11)	0.20610 (11)	0.5905 (7)	0.0495 (14)	
H9A	0.4180	0.2062	0.5116	0.059*	
H9B	0.5957	0.2058	0.7107	0.059*	
C10	0.7642 (12)	0.17604 (11)	0.5585 (7)	0.0508 (14)	
H10A	0.7842	0.1766	0.4389	0.061*	
H10B	0.9579	0.1757	0.6387	0.061*	
C11	0.6143 (13)	0.14651 (12)	0.5818 (8)	0.0592 (16)	
H11A	0.5946	0.1459	0.7014	0.071*	
H11B	0.4205	0.1468	0.5016	0.071*	
C12	0.7623 (15)	0.11758 (13)	0.5502 (9)	0.080 (2)	
H12A	0.7850	0.1179	0.4325	0.121*	
H12B	0.6483	0.0998	0.5634	0.121*	
H12C	0.9496	0.1163	0.6342	0.121*	

### Atomic displacement parameters (Å^2^)

**Table:**

	*U*^11^	*U*^22^	*U*^33^	*U*^12^	*U*^13^	*U*^23^
Br1	0.0519 (3)	0.0577 (3)	0.0587 (3)	0.0057 (6)	0.0134 (2)	0.0063 (5)
O1	0.078 (3)	0.085 (4)	0.069 (3)	0.006 (3)	0.025 (3)	0.006 (3)
N1	0.051 (3)	0.038 (3)	0.056 (3)	0.006 (2)	0.005 (2)	0.0026 (19)
C1	0.050 (4)	0.032 (3)	0.067 (5)	0.001 (3)	0.017 (3)	0.004 (3)
C2	0.052 (3)	0.036 (3)	0.057 (4)	−0.001 (3)	0.016 (3)	0.000 (2)
C3	0.053 (4)	0.043 (3)	0.059 (4)	0.001 (3)	0.027 (3)	0.007 (3)
C4	0.047 (3)	0.039 (3)	0.056 (4)	0.000 (3)	0.017 (3)	0.001 (3)
C5	0.057 (4)	0.041 (3)	0.052 (3)	0.001 (3)	0.024 (3)	−0.005 (3)
C6	0.053 (3)	0.044 (3)	0.049 (3)	−0.003 (3)	0.013 (3)	0.000 (3)
C7	0.048 (3)	0.036 (3)	0.056 (3)	−0.001 (3)	0.020 (3)	0.001 (3)
C8	0.050 (3)	0.042 (3)	0.055 (3)	−0.003 (3)	0.017 (3)	−0.001 (3)
C9	0.051 (4)	0.046 (3)	0.056 (4)	−0.007 (3)	0.021 (3)	−0.001 (3)
C10	0.058 (4)	0.048 (3)	0.047 (3)	0.000 (3)	0.015 (3)	0.001 (3)
C11	0.073 (4)	0.041 (3)	0.067 (4)	−0.011 (3)	0.026 (3)	−0.001 (3)
C12	0.104 (5)	0.041 (4)	0.097 (5)	0.003 (4)	0.028 (4)	−0.007 (3)

### Geometric parameters (Å, °)

**Table:**

O1—H1	0.83 (4)	C6—C7	1.516 (6)
O1—H2	0.97 (4)	C6—H6A	0.9700
N1—C1	1.468 (7)	C6—H6B	0.9700
N1—H1A	0.8900	C7—C8	1.490 (6)
N1—H1B	0.8900	C7—H7A	0.9700
N1—H1C	0.8900	C7—H7B	0.9700
C1—C2	1.501 (7)	C8—C9	1.509 (7)
C1—H1D	0.9700	C8—H8A	0.9700
C1—H1E	0.9700	C8—H8B	0.9700
C2—C3	1.522 (6)	C9—C10	1.532 (7)
C2—H2A	0.9700	C9—H9A	0.9700
C2—H2B	0.9700	C9—H9B	0.9700
C3—C4	1.514 (7)	C10—C11	1.490 (7)
C3—H3A	0.9700	C10—H10A	0.9700
C3—H3B	0.9700	C10—H10B	0.9700
C4—C5	1.506 (6)	C11—C12	1.481 (8)
C4—H4A	0.9700	C11—H11A	0.9700
C4—H4B	0.9700	C11—H11B	0.9700
C5—C6	1.496 (6)	C12—H12A	0.9600
C5—H5A	0.9700	C12—H12B	0.9600
C5—H5B	0.9700	C12—H12C	0.9600
			
H1—O1—H2	91 (5)	C5—C6—H6B	108.3
C1—N1—H1A	109.5	C7—C6—H6B	108.3
C1—N1—H1B	109.5	H6A—C6—H6B	107.4
H1A—N1—H1B	109.5	C8—C7—C6	114.3 (4)
C1—N1—H1C	109.5	C8—C7—H7A	108.7
H1A—N1—H1C	109.5	C6—C7—H7A	108.7
H1B—N1—H1C	109.5	C8—C7—H7B	108.7
N1—C1—C2	111.6 (5)	C6—C7—H7B	108.7
N1—C1—H1D	109.3	H7A—C7—H7B	107.6
C2—C1—H1D	109.3	C7—C8—C9	114.9 (4)
N1—C1—H1E	109.3	C7—C8—H8A	108.5
C2—C1—H1E	109.3	C9—C8—H8A	108.5
H1D—C1—H1E	108.0	C7—C8—H8B	108.5
C1—C2—C3	112.4 (4)	C9—C8—H8B	108.5
C1—C2—H2A	109.1	H8A—C8—H8B	107.5
C3—C2—H2A	109.1	C8—C9—C10	115.3 (4)
C1—C2—H2B	109.1	C8—C9—H9A	108.5
C3—C2—H2B	109.1	C10—C9—H9A	108.5
H2A—C2—H2B	107.9	C8—C9—H9B	108.5
C4—C3—C2	113.2 (4)	C10—C9—H9B	108.5
C4—C3—H3A	108.9	H9A—C9—H9B	107.5
C2—C3—H3A	108.9	C11—C10—C9	115.3 (4)
C4—C3—H3B	108.9	C11—C10—H10A	108.5
C2—C3—H3B	108.9	C9—C10—H10A	108.5
H3A—C3—H3B	107.8	C11—C10—H10B	108.5
C5—C4—C3	114.6 (4)	C9—C10—H10B	108.5
C5—C4—H4A	108.6	H10A—C10—H10B	107.5
C3—C4—H4A	108.6	C12—C11—C10	114.8 (5)
C5—C4—H4B	108.6	C12—C11—H11A	108.6
C3—C4—H4B	108.6	C10—C11—H11A	108.6
H4A—C4—H4B	107.6	C12—C11—H11B	108.6
C6—C5—C4	115.1 (4)	C10—C11—H11B	108.6
C6—C5—H5A	108.5	H11A—C11—H11B	107.5
C4—C5—H5A	108.5	C11—C12—H12A	109.5
C6—C5—H5B	108.5	C11—C12—H12B	109.5
C4—C5—H5B	108.5	H12A—C12—H12B	109.5
H5A—C5—H5B	107.5	C11—C12—H12C	109.5
C5—C6—C7	115.9 (4)	H12A—C12—H12C	109.5
C5—C6—H6A	108.3	H12B—C12—H12C	109.5
C7—C6—H6A	108.3		
			
N1—C1—C2—C3	179.9 (4)	C5—C6—C7—C8	178.9 (4)
C1—C2—C3—C4	170.6 (5)	C6—C7—C8—C9	−179.7 (5)
C2—C3—C4—C5	−178.7 (4)	C7—C8—C9—C10	178.9 (4)
C3—C4—C5—C6	177.0 (5)	C8—C9—C10—C11	−179.1 (5)
C4—C5—C6—C7	−178.4 (4)	C9—C10—C11—C12	179.9 (5)

### Hydrogen-bond geometry (Å, °)

**Table:**

*D*—H···*A*	*D*—H	H···*A*	*D*···*A*	*D*—H···*A*
N1—H1A···Br1^i^	0.89	2.53	3.380 (4)	159
N1—H1B···Br1	0.89	2.47	3.340 (4)	166
N1—H1C···O1	0.89	1.97	2.834 (7)	165
O1—H1···Br1^ii^	0.83 (4)	2.76 (6)	3.329 (5)	128 (6)
O1—H2···Br1^iii^	0.97 (4)	2.49 (5)	3.361 (5)	149 (5)

Symmetry codes: (i) *x*+1, −*y*+1, *z*+1/2; (ii) *x*, −*y*+1, *z*−1/2; (iii) *x*+1, −*y*+1, *z*−1/2.
